# Draft Genome Sequence of *Brevibacillus* sp. Strain LEMMJ03, Isolated from an Antarctic Volcano

**DOI:** 10.1128/MRA.00921-19

**Published:** 2019-10-03

**Authors:** Júnia Schultz, René Kallies, Ulisses Nunes da Rocha, Alexandre Soares Rosado

**Affiliations:** a Laboratory of Molecular Microbial Ecology, Institute of Microbiology, Federal University of Rio de Janeiro, Rio de Janeiro, Brazil; b Department of Environmental Microbiology, Helmholtz Centre for Environmental Research–UFZ, Leipzig, Germany; c Department of Land, Air, and Water Resources, University of California—Davis, Davis, California, USA; Broad Institute

## Abstract

Here, we announce the draft genome sequence of Brevibacillus sp. strain LEMMJ03, isolated from Whalers Bay sediment (Deception Island, Antarctica). In total, 4,500 coding sequences (CDS), among those 102 coding for tRNAs and 5 for noncoding RNAs (ncRNAs), were predicted from the 4.64-Mb genome. Predicted functions were for bacteriocin and degradation of aromatic compounds.

## ANNOUNCEMENT

Members of the genus *Brevibacillus* (phylum Firmicutes) are rod-shaped, Gram-positive, spore-forming, mostly strict aerobes with ubiquitous distribution in soils, including those of Antarctica (1, 2).

Geothermal habitats in Antarctica typically consist of heated mineral soils found near fumarolic vents and are exclusively associated with geologically active volcanoes, such as Deception Island, a marine polar volcano with temperatures ranging from 0 to 120°C and gradients of salinity and geochemistry (2, 3). These unique characteristics make the thermophiles of Antarctica an untapped resource for bioprospecting new enzymes, cosmetical products, and other biotechnological applications (4, 5).

*Brevibacillus* sp. strain LEMMJ03 was isolated from a thermophilic sediment (50°C) collected near a fumarole vent in Whalers Bay (62°58′788″S, 60°33′464″W), Deception Island, Antarctica. Briefly, the sediment (500 g) was collected (depth, 0 to 5 cm), placed in a sterile plastic bag, and stored at 4°C. For bacterial isolation, the sample was homogenized, and 10 g was added to 90 ml of saline (0.85% NaCl) in a flask with glass beads and kept stirring at 2 × *g* for 2 h. Serial 10-fold dilutions were prepared, in which 0.1 ml of each dilution was spread over Bacillus schlegelii heterotrophic medium (DSMZ 260) plates and incubated at 55°C for 48 h.

A single colony was isolated, and genomic DNA extraction was performed using a Wizard genomic DNA purification kit (Promega, Madison, WI, USA) and quantified using a Qubit fluorometer (Thermo Fisher Scientific, Waltham, MA, USA). For the genome sequencing on an Illumina MiSeq platform, a NEBNext Ultra II FS DNA library kit (New England Biolabs, Ipswich, MA, USA) was used to prepare a paired-end 301-bp library, following the manufacturer’s instructions. Sickle (6), with a Phred quality score of >30, was used for the quality control and to trim the sequences. *De novo* assembly was performed using SPAdes (7), and CheckM and RefineM (8) were used for the quality check and to provide completeness and contamination information. Annotation was carried out with the NCBI Prokaryotic Genome Annotation Pipeline (PGAP) (9) and on the Rapid Annotations using Subsystems Technology (RAST) server (10). Default parameters were used for all software.

The draft assembly of *Brevibacillus* sp. LEMMJ03 resulted in a length of 4,646,831 bp with 53× coverage, 98.49% completeness, 1.51% contamination, 142 contigs, and a GC content of 58.7%. Annotation by PGAP predicted, in total, 102 tRNA genes, 5 noncoding RNA (ncRNA) genes, 34 rRNA genes, and 4,500 coding sequences (CDS). The annotation by RAST identified 29 genes for metabolism of central aromatic intermediates and peripheral pathways for catabolism of aromatic compounds, such as those for naphthalene, anthracene, toluene, and xylene degradation. No resistance genes were identified by ResFinder 2.1. (11). In addition, genes coding for potential production of bacteriocin and ectoine were detected with antiSMASH (12), highlighting the biotechnological interest in strain LEMMJ03 for bioremediation of environments contaminated with hydrocarbons and for new antimicrobial products.

### Data availability.

This whole-genome shotgun project has been deposited at DDBJ/ENA/GenBank under the accession number VKJN00000000. The version described in this paper is VKJN01000000. Raw data are available in the NCBI Sequence Read Archive under the accession number SRR10040619, which is part of BioProject number PRJNA554144.
